# Chronic Obstructive Pulmonary Disease Patients With Community‐Acquired Pneumonia on Inhaled Corticosteroid Therapy: A Comprehensive Analysis of Risk Factors, Disease Burden, and Prevention Strategies

**DOI:** 10.1002/hsr2.70395

**Published:** 2025-01-26

**Authors:** Muhammad Muneeb Hassan, Sheikh Muhammad Sikandar, Farrukh Jamal, Muhammad Ameeq, Alpha Kargbo

**Affiliations:** ^1^ Department of Statistics The Islamia University of Bahawalpur Bahawalpur Pakistan; ^2^ DHQ Hospital Muzaffargarh Muzaffargarh Punjab Pakistan; ^3^ Department of Physical and Natural Sciences Brikama Campus University of the Gambia Serrekunda Gambia

**Keywords:** community‐acquired pneumonia, COPD, coronavirus disease 2019, SARS‐CoV‐2, smoking history

## Abstract

**Background:**

Chronic obstructive pulmonary disease (COPD) patients commonly exhibit significant morbidity and experience a diminished quality of life. Since there has been no prior research on pneumonia in our study population, we carried out this study to learn more about the situation.

**Methods:**

A retrospective analysis of 912 COPD patients with CAP who were receiving ICS treatment at the DHQ Hospital in Muzaffargarh, Punjab, Pakistan was conducted. Study began in February 2022 and ended in February 2023. Using multinomial logistic regression, the odds ratio and relative risk and Kaplan–Meier curves showed time‐to‐death and recovery by COPD status.

**Results:**

Patients with COPD having a smoking history from 25 pack years and above had 22.791 higher odds of CAP (95% CI: 20.413–31.515), 21.527 higher odds of HTN (95% CI: 12.323–57.103), 16.955 higher odds of diabetes (95% CI: 22.954–29.331), and 13.964 higher odds of death in severity without COVID‐19 vaccination (95% CI: 5.988–32.561) compared to patients with COPD having a smoking history from 10 to 15 pack years.

**Conclusion:**

COPD patients with a shorter ICS duration had a lower CAP risk, and vice versa, while vaccinated patients had a less severe disease as compared to non‐vaccinated patients.

**Abbreviations**
CAP: community acquired pneumonia
COPD: chronic obstructive pulmonary disease
DM: diabetes mellitus
HTN: hypertension
ICS: inhaled corticosteroids
LAMA: left against medical advice
PHC: Punjab Health Council
SARS‐CoV‐2: severe acute respiratory syndrome coronavirus‐2

## 1 Introduction

There is indeed a complex and potentially growing burden on health services all over the world due to chronic obstructive pulmonary disease (COPD), which is currently ranked third on the list of major factors contributing to mortality. More than 250 million people right now suffer with COPD, and it predicts that by 2060, COPD will be responsible for more than 6 million deaths annually, making it the fifth leading cause of death worldwide. In Pakistan, the prevalence of COPD among adults aged 40 and older was found to be 2.1% [1, 2].

COPD is defined by chronic respiratory system symptoms (shortening of breath, coughing with expectoration, and sputum) due to abnormalities of either the airways (bronchitis) or alveoli (emphysema) that cause persistence and progressive airflow obstruction that is not or only partially reversible with bronchodilators. COPD causes both airway and systemic inflammation [3].

In countries with low and middle incomes, an astonishingly high percentage of people under the age of seventy die from COPD [1, 4]. Community‐acquired pneumonia (CAP) is a major contributor to COPD deaths, and the GOLD guidelines emphasis the role of inhaled corticosteroids (ICS) in the management of COPD. Therefore, preventing respiratory infections is an important part of managing COPD and lowering the risk of exacerbations [5]. The GOLD guidelines recommended pneumococcal polysaccharide, influenza, and COVID‐19 vaccinations [6, 7]. The combination of ICS and LABA is associated with lower FEV1 decline, improved lung function, reduced exacerbations, and improved overall health status.

However, Long‐term use of ICS, particularly in patients with comorbidities such as diabetes and hypertension, increases the risk of developing CAP [8]. Several studies have demonstrated a link between the use of ICS and an increased risk of CAP and hospitalization among people with COPD, especially those with severe disease [9, 10]. Some studies suggest that prolonged ICS use may be associated with an increased risk of pneumonia, particularly in patients with comorbidities. Both vaccinated and non‐vaccinated groups were considered [6, 11].

The purpose of this study was to determine the safety and efficacy of ICS, the likelihood of exacerbations, and deaths related to CAP. There has been no study in the last 5 years in our area to determine the incidence of CAP in COPD patients receiving ICS therapy, so this study aimed to fill that void [12, 13, 14, 15]. As a starting point, our proposed study will report on the current scope of the problem and provide demographic data about our population. Clinicians will be able to optimize the duration of ICS, foresee CAP, and treat it promptly [16, 17, 18]. This will reduce the morbidity and mortality associated with the disease.

## 2 Materials and Methods

A retrospective study was conducted at the DHQ Hospital Muzaffargarh covering rural and urban areas of four subdistricts to screen 912 male and female patients for COPD with pneumonia, despite the fact that the total population of District Muzaffargarh was 43,48,549 [19]. According to the statistical officer at the DHQ Hospital Muzaffargarh, patient records remained in 2021–22; the total number of OPD, indoor, COPD with pneumonia, and COPD (vaccinated and non‐vaccinated patients of COVID–19) was 28,731, 12,342, 1123, and 1029, respectively.

COPD patients were diagnosed based on spirometry measurements suggestive of airflow limitation, that is, a ratio of FEV1/FVC less than 0.7 of the predicted value that is only partially reversible with an inhaled bronchodilator. COPD patients with a history of more than 10 pack years of smoking were included. The majority of study participants were taking either fluticasone and salmeterol or budesonide and formoterol, fluticasone dosages of 500–1000 mg per day, and/or budesonide dosages of 400–800 μg per day throughout the course of study. Pneumonia was diagnosed based on clinical findings of a history of fever, worsening cough, shortness of breath, and a change in color or quantity of sputum, along with chest X‐rays showing consolidation. We also included COVID‐19 (vaccinated/unvaccinated) cases reported from January 2021 to December 2022 using a simple random technique. To confirm the COVID‐19 cases, laboratories certified by the Punjab Health Council (PHC) and the Research Institute of Nishtar Medical College and University Multan used real‐time reverse transcription polymerase chain reaction. This study began in February 2022 and ended in February 2023; data collection ended in December 2022. The Kaplan–Meier curves were used to describe time‐to‐death and time‐to‐recovery based on COPD status. The SPSS‐22, Math Type, and R were used to conduct all statistical analyses. The study followed the Declaration of Helsinki guidelines and was approved on February 8, 2023, with reference No. 154‐58/DHQ, in DHQ Hospital Muzaffargarh, Punjab, Pakistan.

### 2.1 Multinomial Logistic Regression Model for COPD With CAP Patients

Multinomial logistic regression is used when the result can have more than two categories that are not in ordered [20]. We include all related variables in equation according to our criteria.

$$\begin{array}{l} Y=\left(\text{Area}/\text{Age}\right)+\beta_{1}\left(\text{DM}\right)+\beta_{2}\left(\text{HTN}\right)+\beta_{3}\left(\text{obesity}\right)+\beta_{4}\left(\text{Inhaler}\;\text{duration}\right)+\beta_{5}\left(\text{dyslipidemia}\right)+\beta_{6}(\text{CAP})+\beta_{7}\left(\text{severity}\;\text{with}\;\text{vaccination}\right)+\beta_{8}\left(\text{severity}\;\text{without}\;\text{vaccination}\right). \end{array}$$

### 2.2 Hypothesis

These are eight hypotheses drawn from COPD patients who had pneumonia.

**Hc**_**1**_ = diabetes mellitus (DM) has a significant impact on COPD.
**Hc**_**2**_ = hypertension (HTN) has a significant impact on COPD.
**Hc**_**3**_ = obesity has a significant impact on COPD.
**Hc**_**4**_ = inhaler duration has a significant impact on COPD.
**Hc**_**5**_ = dyslipidemia has a significant impact on COPD.
**Hc**_**6**_ = CAP has a significant impact on COPD.
**Hc**_**7**_ = severity with COVID‐19 vaccination has a significant impact on COPD.
**Hc**_**8**_ = severity without COVID‐19 vaccination has a significant impact on COPD.

## 3 Results

A total of 912 COPD patients were hospitalized with CAP, with 790 male and 122 female patients, ranging in age from 32 to more than 52, exhibiting symptoms. To make a suitable group, smoking histories were taken from 1 month to more than 1 year. The patient's death rate ratio is based on independent variables such as diabetes, HTN, obesity, Inhaler duration, dyslipidemia, and CAP, as well as severity with and without COVID‐19 vaccination. All variables were statistically significant at *p* < 0.05, indicating their relevance in assessing pneumonia risk among COPD patients, as shown in Table 1.

**Table 1:** Characteristics table.

	Value (%) Total = 912	Mean (SD)	95% Confidence interval for mean		*p*‐value*
			Lower	Upper	
Gender	—	0.13 (0.341)	0.110–0.160		
Male	790 (86.62)	—	—		0.000
Female	122 (13.37)	—	—		
Age	—	0.30 (0.459)	0.270–0.330		
32–52	638 (69.95)	—	—		0.000
52–72 and above	274 (30.04)	—	—		
Area**	—	0.52 (0.500)	0.480–0.550		
Rural	442 (48.46)	—	—		0.000
Urban	470 (51.35)	—	—		
Muzaffargarh	490 (53.72)				
Kot Addu	113 (12.39)				
Jatoi	211 (23.13)				
Ali Pur	98 (10.74)				
Smoking history in pack (years)	—	2.80 (1.193)	2.730–2.880		
	—	—	—		0.000
10–15	205 (22.47)	—	—		
15–20	140 (15.35)	—	—		
20–25	196 (21.49)	—	—		
25 and above	371 (40.67)	—	—		
SES	—	0.78 (0.415)	0.751–0.810		0.02
Middle income area	201 (22.03)	—	—		
Poor income area	711 (77.96)	—	—		
Inhaler duration	—	3.22 (1.072)	3.160–3.290		0.049
1–3 months	22 (2.41)	—	—		
3–6 months	236 (25.87)	—	—		
6–9 months	311 (34.10)	—	—		
9–12 months	201 (22.03)	—	—		
More than 1 year	142 (15.57)	—	—		
DM	236 (25.87)	0.26 (0.438)	0.230–0.290		0.000
HTN	618 (67.76)	0.68 (0.468)	0.650–0.710		0.000
Obesity	201 (22.03)	0.22 (0.415)	0.190–0.250		0.000
Dyslipidemia	413 (45.85)	0.45 (0.498)	0.420–0.490		0.019
CAP	130 (14.25)	0.14 (0.335)	0.120–0.170		0.000
Severity with vaccination	795 (87.17)	0.87 (0.335)	0.850–0.890		0.008
Severity without vaccination	173 (18.96)	0.19 (0.392)	0.160–0.120		0.000
Event (death)	192 (21.05)	0.03 (0.181)	0.020–0.050		0.000

* *p*‐value (< 0.05) is significant.

** Area covered for the patient of COPD patients with community‐acquired pneumonia, namely subdistricts Muzaffargarh, Kot addu, Ali pur, and Jattoi.

It demonstrates that all of the regression coefficients in the model are equal to zero under the null hypothesis. When compared to the alpha level of 0.05, this *p*‐value is determined to be lower than the alpha level. As a result, the model is deemed significant. The Cox and Snell $R^{2}$, Nagelkerke, and McFadden have a value of 0.515, 0.703, and 0.411 which means that the proportion of variance of disease COPD having a smoking history is 51%, 70%, and 41%, which is explained by independent variables like DM, HTN, obesity, Inhaler duration, dyslipidemia, CAP, vaccination of COVID‐19 and severity. We use the Nagelkerke for the best measurement by the model with a large $R^{2}$ at 70.3%, as shown in Table 2.

**Table 2:** Model fitting information.

Model	Model fitting criteria			Likelihood ratio tests		
	AIC	BIC	−2 Log Likelihood	Chi‐square	*df*	Significance
Intercept only	1326.14	1340.50	1320.13	—	—	—
Final	714.16	844.183	660.161	659.978	24	.00
Goodness‐of‐fit						
Pearson	—	—	—	459.400	468	.00
Deviance	—	—	—	439.068	468	.00
Pseudo *R* ^2^						
Cox and Snell			Nagelkerke		McFadden	
0.515			0.703		0.411	

### 3.1 Smoking History From 15 to 20 Pack Years Relative to the Smoking History From 10 to 15 Pack Years

Results of estimates represent that the smoking history from 15 to 20 years relative to the smoking history from 10 to 15 years when the predicted variables in the model are evaluated as zero. The logit of achieving these results is −1.160. When one unit increases in the DM, HTN, Inhaler duration, dyslipidemia, Severity with vaccination of COVID‐19 and Severity without vaccination of COVID‐19 for smoking history from 15 to 20 years relative to the smoking history from 10 to 15 years, then the multinomial log odd increase by 5.62, 18.066, 0.008, 12.880, 0.007, and 0.728, holding all other variables constant. While, when one unit increases in the CAP and obesity, then the multinomial log odd decreases by 5.610 and 1.170, holding all other variables constant.

If, on the other hand, units increase in DM, HTN, obesity, Inhaler duration, dyslipidemia, CAP, severity with COVID‐19 vaccination, and severity without COVID‐19 vaccination, the relative risk for performing a smoking history from 15 to 20 years relative to a smoking history from 10 to 15 years would be expected to increase by a factor of 2.225, 0.936, 4.090, 0.992, 0.415, 0.310, 4.515, and 2.071 times, respectively, when all other variables are held constant as shown in (Table 3).

**Table 3:** Estimates of smoking history based on age factors using multinomial logistic regression.

Smoking*	*Β*	Standard error	Wald	*df*	Significance	Exp (*B*)	95% Confidence interval for Exp (*β*)	
							Lower Bound	Upper Bound
Smoking History from 15–20 pack years								
Intercept	−1.160	0.639	3.296	1	0.069	—	—	—
DM	5.620	0.000	—	1	—	2.225	2.225	2.225
HTN	18.066	0.404	0.026	1	0.871	0.936	0.424	2.068
Obesity	−5.610	0.000	—	1	—	4.090	4.090	4.090
Inhaler duration	0.008	0.110	0.006	1	0.939	0.992	0.799	1.231
Dyslipidemia	12.880	0.419	4.417	1	0.036	0.415	0.183	0.942
CAP	−1.170	0.484	5.841	1	0.016	0.310	0.120	0.802
Severity with vaccination**	0.007	0.509	8.769	1	0.003	4.515	1.665	12.246
Severity without vaccination***	0.728	0.454	2.567	1	0.109	2.071	0.850	5.046
Smoking history from 20–25 pack years								
Intercept	0.577	0.521	1.228	1	0.268	—	—	—
DM	4.103	1.025	16.017	1	0.001	12.533	8.115	21.537
HTN	20.549	0.349	19.715	1	0.001	4.708	2.376	9.328
Obesity	−3.469	0.303	2.387	1	0.122	0.626	0.345	1.134
Inhaler duration	3.176	0.101	3.006	1	0.083	0.839	0.687	1.023
Dyslipidemia	16.969	0.353	31.122	1	0.001	0.140	0.070	0.279
CAP	1.132	0.282	16.071	1	0.001	3.102	1.783	5.395
Severity with vaccination**	1.585	0.382	2.347	1	0.126	0.557	0.264	1.178
Severity without vaccination***	2.067	0.416	0.026	1	0.871	0.935	0.414	2.112
Smoking history from 25 pack years and above								
Intercept	−3.903	0.672	33.728	1	0.001	—	—	—
DM	11.118	1.012	25.555	1	0.001	16.955	22.954	29.331
HTN	32.278	0.391	70.230	1	0.001	21.527	12.323	57.103
Obesity	−0.001	0.273	4.365	1	0.037	1.769	1.036	3.020
Inhaler duration	6.036	0.102	0.125	1	0.724	2.965	0.790	3.177
Dyslipidemia	22.911	0.338	32.019	1	0.001	8.148	0.076	10.287
CAP	33.235	0.331	0.501	1	0.479	22.791	20.413	31.515
Severity with vaccination**	0.185	0.476	21.036	1	0.001	8.888	3.494	22.608
Severity without vaccination***	8.636	0.432	37.252	1	0.001	13.964	5.988	32.561

* The reference category is: smoking history from 10–15 pack years.

** The patient is vaccinated with COVID‐19, and their health condition shows severity.

*** The patient is not vaccinated with COVID‐19, and their health condition shows severity.

### 3.2 Smoking History From 20 to 25 Pack Years Relative to the Smoking History From 10 to 15 Pack Years

According to the results when one unit increases in the DM, HTN, Inhaler duration, dyslipidemia, CAP, severity with vaccination and Severity without vaccination for smoking history from 20 to 25 years relative to the smoking history from 10 to 15 years, then the multinomial log odd increases by 4.103, 20.549, 3.176, 16.969, 1.132, 1.585, and 2.067 holding all other variables constant. While one unit increases in obesity, the multinomial log odd decreases by 3.469, holding all other variables constant.

Alternatively, if units increase in DM, HTN, obesity, Inhaler duration, dyslipidemia, CAP, severity with COVID‐19 vaccination, and severity without COVID‐19 vaccination, the relative risk for performing a smoking history from 20 to 25 years relative to a smoking history from 10 to 15 years would be expected to increase by a factor of 12.533, 4.708, 0.626, 0.839, 0.140, 3.102, 0.557, and 0.935 times, respectively, when all other variables are held constant as shown in (Table 3).

### 3.3 Smoking History From 25 Pack Years and Above Relative to the Smoking History From 10 to 15 Pack Years

Multinomial log odd increases by 11.118, 32.278, 6.036, 22.911, 33.235 0.185, and 8.636 respectively, for increases of one unit in DM, HTN, Inhaler duration, dyslipidemia, CAP, severity with vaccination and severity without vaccination for smoking histories from 25 years and above compared to smoking histories from 10 to 15 years. While in obesity reduce by 0.001.

Alternatively, if units increase in DM, HTN, obesity, Inhaler duration, dyslipidemia, CAP, severity with COVID‐19 vaccination, and severity without COVID‐19 vaccination, the relative risk for performing a smoking history from 25 years and above relative to a smoking history from 10 to 15 years would be expected to increase by a factor of 16.955, 21.527, 1.769, 2.965, 8.148, 22.791, 8.888, and 13.964 times, respectively, when all other variables are held constant as shown in Table 3.

**Figure 1:**
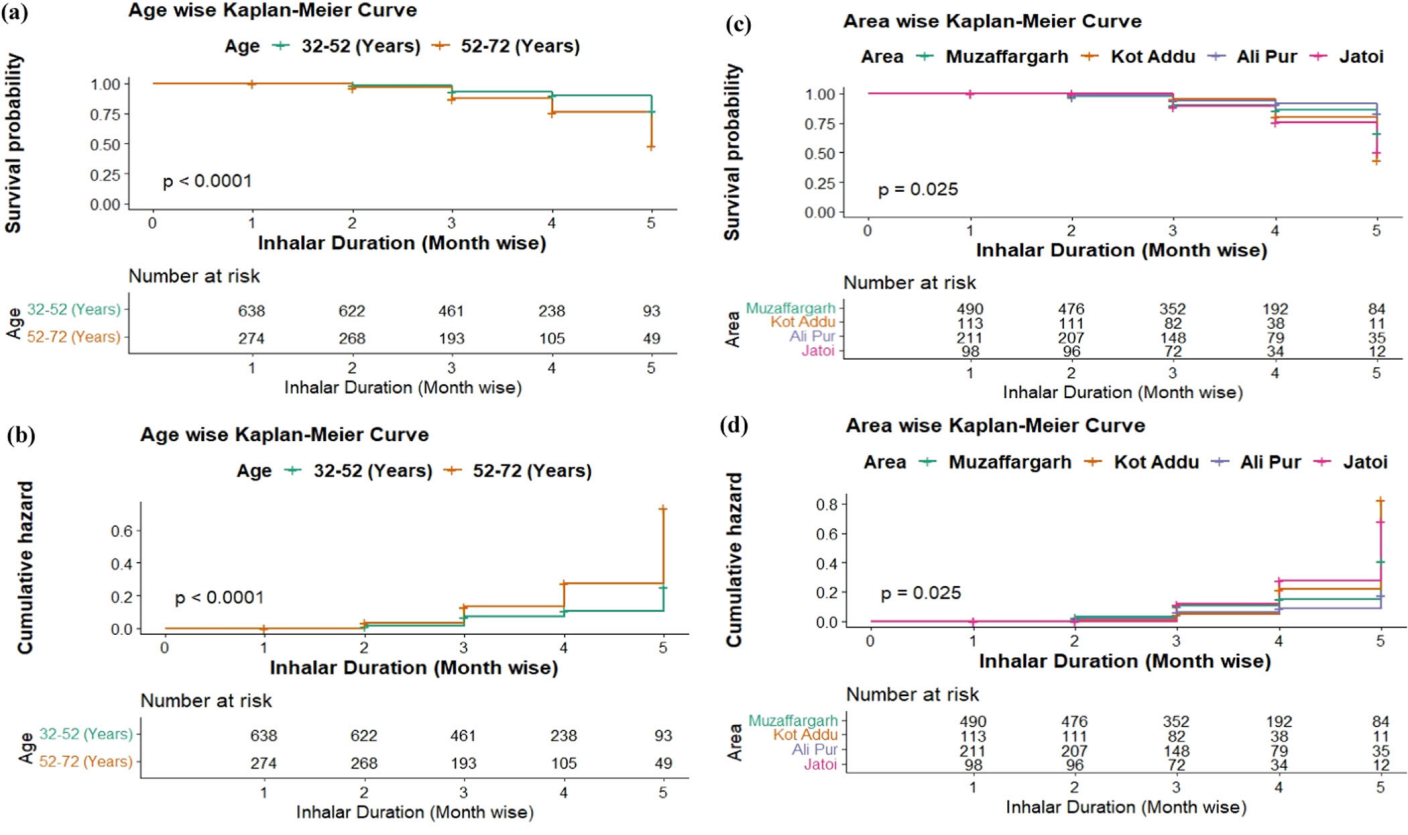
Survival analysis. (a) Age wise Kaplan–Meier curve, time‐to‐death, and survival evaluated the survival function. (b) Age wise Kaplan–Meier curve, time‐to‐death, and survival evaluated the hazard function. (c) Area wise Kaplan–Meier curve, time‐to‐death, and survival evaluated the survival function. (d) Area wise Kaplan–Meier curve, time‐to‐death and survival evaluated the hazard function. Inhaler Duration coded in (a)–(d): 1–3 months as 1, 3–6 months as 2, 6–9 months as 3, 9–12 months as 4, and more than 1 year as 5.

Kaplan–Meier curves depicted the results pattern of the survival and hazard graph proposed for COPD with CAP patients using inhaler with respect to 1 month to more than a year in (Figure 1). The survival and hazards graphs in Figure 1a–d provide an overview of the typical progression of patient outcomes. The findings support the hypothesis that COPD and CAP patients who used their inhalers for a year or more had a much lower survival rate.

## 4 Discussion

In our study, we explored that COPD patients who were prescribed ICS for a shorter period of time had a lower risk of developing CAP, as well as the contrary was also true: patients who were prescribed ICS for a longer period of time had a higher risk of developing CAP. Our findings indicate that the risk of developing CAP among COPD patients using ICS is closely linked to the presence of comorbidities such as diabetes, HTN, and smoking history, suggesting that ICS use alone is not the sole contributor but interacts with these factors to exacerbate the risk; while conversely, patients who were prescribed ICS for longer durations demonstrated an elevated risk of developing CAP. This discovery suggests that the duration of ICS use among individuals with COPD may have a notable impact on their susceptibility to CAP. It is crucial to comprehend that ICS are commonly employed in the management of COPD due to their demonstrated efficacy in mitigating airway inflammation and enhancing pulmonary function. Nevertheless, there is evidence suggesting a correlation between the utilization of ICS and a heightened susceptibility to respiratory infections, including CAP. This phenomenon may be attributed to the immunosuppressive effects of ICS, which render individuals more vulnerable to various illnesses. Shorter courses of ICS treatment may reduce the risk of infection, whereas longer courses may increase the risk.

Our study included 912 patients who met the inclusion criteria; 790 (86.62%) were male patients, while 122 (13.37%) were female patients. Various studies have reported a high male predominance in literature. According to our results, DM, HTN, inhaler duration, dyslipidemia, severity with vaccination of COVID‐19, and severity without vaccination of COVID‐19, for smoking histories from 15 to 20 pack years relative to smoking histories from 10 to 15 pack years, show highly significant results. But CAP patients taking ICS and having obesity show low significance. However, DM, HTN, inhaler duration, dyslipidemia, CAP patients taking ICS, severity with vaccination, and severity without vaccination for smoking histories from 25 pack years and above, relative to smoking histories from 10 to 15 pack years, show highly significant results, while obesity shows low significance. If units increase in CAP patients taking ICS, DM, HTN, and severity without COVID‐19 vaccination, the relative risk for smoking history from 25 pack years and above relative to 10–15 pack years would increase by a factor.

In related studies, the secondary goal of the Louisville hospital study was to examine CAP. Patients with COPD and severe acute respiratory syndrome coronavirus‐2 (SARS‐CoV‐2) CAP were more likely to experience a longer hospital stay (15 vs. 5 days, *p* = 0.001), cardiac events (4.98 higher odds; 95% CI: 3.74–6.69), and death (7.31 higher odds; 95% CI: 5.311–15.12) [21]. The Emergency Department of Wan Fang Hospital found that COPD patients with pneumonia had significantly higher CRP levels than those with AECOPD (median: 6.7, IQR: 23.6–2.4) (*p* = 0.001) [22]. On the other hand, an increase in the number of CAP has been reported in conjunction with COPD in recent years, and this has been shown to negatively affect QOL, healthcare utilization, and even survival [23]. Previous research has suggested that administering steroids late in the course of an infection could severely hinder the lung's ability to regain homeostasis. Although it has been hypothesized that using inhaled steroids raises the risk of severe pneumonia in COPD patients, there is no conclusive evidence to support this. Patients with COPD and those without it exhibit dissimilar clinical manifestations of CAP. Patients with severe COPD who are receiving oxygen therapy at home should consider not only *Streptococcus pneumoniae* but also Gram‐negative bacilli and *Pseudomonas aeruginosa* as potential causes of their condition [2].

Our findings indicate that while ICS are effective in managing COPD, their prolonged use may interact with comorbidities such as diabetes and HTN, potentially increasing pneumonia risk. The duration of ICS has a strong correlation with CAP in patients with a smoking history of 15–20 years; with shorter inhaler duration, CAP risk is not increased but rather shows a decreasing trend. Comparatively, patients with a smoking history of 20–25 pack years and a relatively longer ICS duration show a 1.13‐unit increase in CAP frequencies. However, patients with a smoking history of 25 pack years and above had a longer duration of ICS and a dramatically increased CAP ratio of 33.332 units.

Our research also showed that areas with higher COPD prevalence had higher fatality rates. The disease pattern must be overcome with health planning and resource allocation. In many underdeveloped countries with higher COPD severity rates, COVID‐19 cases are quickly increasing. COVID‐19 can be mitigated by COPD management. Several SARS‐CoV‐2 studies have examined the relationship between CAP and COPD. Patients with infections are more likely to decline.

Although SARS‐CoV‐2 indirectly contributes to morbidity and mortality, our findings also demonstrate a direct link between CAP and COPD. Worldwide health systems were impacted by COVID‐19, and COPD mortality rates are predicted to increase as a result of drug shortages and delays in diagnosis and treatment. The inability to pay for medical expenses is another consequence of the healthcare system's accessibility issues, which disproportionately impacts populations who are socially disadvantaged. Our results highlight how crucial it is to maintain COPD patients' vaccinations, and COVID testing services are a top priority, despite the fact that the COVID‐19 pandemic has altered health and social systems. Making inferential comparisons necessitates longer follow‐up studies with a sufficient sample size. According to what we discovered, this study has a sufficient number of participants to examine the relationship between COPD and CAP with a high burden of COVID‐19 cases.

HIV‐positive patients with COPD will also have to manage the consequences of residual confounding. Patients with COPD and no other conditions would have made it easier to rule out other factors, but we did not have any information on HIV‐AIDS in our study, which could have influenced our estimates. People whose dates were missing were not included in the survival analyses. Each time‐to‐event analysis used a different sample size, and statistical analyses were performed to determine how widely dispersed the confounding factors were. There may also be gaps in the information we have regarding the two most important outcomes (survival and mortality). This was remedied by excluding patients who did not reach an outcome by the end of the follow‐up period from the survival analysis (LAMA cases).

## 5 Conclusion

This study found that DM, HTN, CAP, duration of ICS and vaccination in patients of COPD are the most significant contributors towards disease morbidity and mortality. Controlling these factors eliminates or reduces the disease burden. Patients with DM, HTN, CAP and severity without vaccination had a greater risk of death and a lower chance of recovery. The study concluded that while minimizing ICS duration may reduce the incidence of CAP, managing comorbid conditions like diabetes, HTN, and smoking habits is equally important for reducing CAP risk in COPD patients. However, in patients who had shorter smoking histories and brief ICS use, CAP risk was significantly decreased. Long‐term (> 6 months) ICS use significantly increased the risk of CAP; therefore, vaccination had a greater impact on the severity of CAP, being more severe in non‐vaccinated patients as compared to vaccinated patients. The results of our study have the potential to improve the delivery of health care to people with COPD and to target those in maximum need of assistance with disease management and prevention strategies.

## Author Contributions

**Muhammad Muneeb Hassan:** conceptualization, data analysis, coding, graph making, and final approval of the version to be published. **Sheik Muhammad Sikandar:** accuracy of clinical data, editing, proofreading, data interpretation, writing, and final approval for publication. **Farrukh Jamal:** proofreading, data interpretation, writing, and final approval for publication. **Muhammad Ameeq:** proofreading, data interpretation, writing, and final approval for publication. **Alpha Kargbo:** editing, proofreading, data interpretation, writing, and final approval for publication. All authors contributed to interpreting data, drafting the manuscript, and critically revising the manuscript for intellectual content, all authors approved of the published version.

## Consent

The authors have nothing to report.

## Conflicts of Interest

The authors declare no conflicts of interest.

### 1 Transparency Statement

The authors affirm that all listed contributors made substantial contributions to the development of this manuscript. Each author has reviewed and approved the final version of the article. The recent change in authorship reflects contributions made during the later stages of the study, with specific roles clarified as follows: Sheikh Muhammad Sikandar provided essential support in data collection and management, ensuring the accuracy of clinical data; Farrukh Jamal guided the statistical analysis and contributed to the interpretation of results. This revised author list accurately represents each contributor's involvement and contribution, and all authors declare no conflicts of interest related to this change.

## Data Availability

The data that support the findings of this study are available on request from the corresponding author.
